# Preferential expression of *SCN1A* in GABAergic neurons improves survival and epileptic phenotype in a mouse model of Dravet syndrome

**DOI:** 10.1007/s00109-023-02383-8

**Published:** 2023-10-11

**Authors:** Ana Ricobaraza, Maria Bunuales, Manuela Gonzalez-Aparicio, Saja Fadila, Moran Rubinstein, Irene Vides-Urrestarazu, Julliana Banderas, Noemi Sola-Sevilla, Rocio Sanchez-Carpintero, Jose Luis Lanciego, Elvira Roda, Adriana Honrubia, Patricia Arnaiz, Ruben Hernandez-Alcoceba

**Affiliations:** 1 Gene Therapy and Regulation of Gene Expression Program, CIMA, University of Navarra, CIMA, Av. Pio XII 55, E-31008 Pamplona, Spain; 2 Sackler Faculty of Medicine, Goldschleger Eye Research Institute, Tel Aviv University, Tel Aviv, Israel; 3 Department of Human Molecular Genetics and Biochemistry, Sackler Faculty of Medicine, Tel Aviv University, Tel Aviv, Israel; 4 Sagol School of Neuroscience, Tel Aviv University, Tel Aviv, Israel; 5 University Clinic of Navarra, Dravet Syndrome Unit, Pediatric Neurology Unit, IdiSNA, Navarra Institute for Health Research, Pamplona, Spain; 6 Department of Neuroscience, CIMA, University of Navarra, Pamplona, Spain; 7 Instituto de Investigación Sanitaria de Navarra (IdiSNA), Pamplona, Spain; 8 Centro de Investigación Biomédica en Red de Enfermedades Neurodegenerativas (CiberNed), Madrid, Spain

**Keywords:** *SCN1A*, Nav1.1, Dravet syndrome, Epileptic encephalopathy, Adenoviral vector, ErbB4, GABAergic neuron, DP3V, *Dlx*

## Abstract

**Abstract:**

The *SCN1A* gene encodes the alpha subunit of a voltage-gated sodium channel (Na_v_1.1), which is essential for the function of inhibitory neurons in the brain. Mutations in this gene cause severe encephalopathies such as Dravet syndrome (DS). Upregulation of *SCN1A* expression by different approaches has demonstrated promising therapeutic effects in preclinical models of DS. Limiting the effect to inhibitory neurons may contribute to the restoration of brain homeostasis, increasing the safety and efficacy of the treatment. In this work, we have evaluated different approaches to obtain preferential expression of the full *SCN1A* cDNA (6 Kb) in GABAergic neurons, using high-capacity adenoviral vectors (HC-AdV). In order to favour infection of these cells, we considered ErbB4 as a surface target. Incorporation of the EGF-like domain from neuregulin 1 alpha (NRG1α) in the fiber of adenovirus capsid allowed preferential infection in cells lines expressing ErbB4. However, it had no impact on the infectivity of the vector in primary cultures or in vivo. For transcriptional control of transgene expression, we developed a regulatory sequence (DP3V) based on the Distal-less homolog enhancer (*Dlx*), the vesicular GABA transporter (*VGAT*) promoter, and a portion of the *SCN1A* gene. The hybrid DP3V promoter allowed preferential expression of transgenes in GABAergic neurons both in vitro and in vivo. A new HC-AdV expressing *SCN1A* under the control of this promoter showed improved survival and amelioration of the epileptic phenotype in a DS mouse model. These results increase the repertoire of gene therapy vectors for the treatment of DS and indicate a new avenue for the refinement of gene supplementation in this disease.

**Key messages:**

- Adenoviral vectors can deliver the *SCN1A* cDNA and are amenable for targeting.
- An adenoviral vector displaying an ErbB4 ligand in the capsid does not target GABAergic neurons.
- A hybrid promoter allows preferential expression of transgenes in GABAergic neurons.
- Preferential expression of *SCN1A* in GABAergic cells is therapeutic in a Dravet syndrome model.

**Supplementary Information:**

The online version contains supplementary material available at 10.1007/s00109-023-02383-8.

## Introduction

The *SCN1A* gene encodes the alpha subunit of a voltage-gated sodium channel (Na_v_1.1), which is expressed preferentially, but not exclusively, in GABAergic neurons of the central nervous system (CNS) [1, 2]. The role of Na_v_1.1 as initiator and propagator of action potentials is especially important in inhibitory interneurons such as those expressing parvalbumin (PV) and somatostatin (ST) [3, 4]. Mutations in *SCN1A* are associated with different neurological diseases, from the relatively mild genetic epilepsy with febrile seizures plus (GEFS^+^) to the severe myoclonic epilepsy in infancy (SMEI), also known as Dravet syndrome (DS), OMIM 607208 [5]. More than 80% of DS cases are caused by de novo heterozygous mutations of *SCN1A*. The initial manifestations of DS start during the first year of life and consist of frequent, drug-resistant, and often prolonged seizures, typically triggered by hyperthermia. Cognitive, motor, and behavioural alterations appear at later stages with different degrees of severity [6, 7]. The risk of sudden unexpected death in epilepsy (SUDEP) remains high at all ages. The overall mortality of DS in industrialized countries is 10–15%, resulting from SUDEP or status epilepticus (SE) in 50 and 35% of cases, respectively [8]. The most accepted physiopathology of DS is based on a disequilibrium between excitatory and inhibitory signals in the brain. Results obtained in mouse models indicate that *SCN1A* haploinsufficiency impairs the function of GABAergic interneurons [9–13]. In fact, the DS phenotype is exacerbated when mutations are restricted to inhibitory neurons, suggesting that haploinsufficiency in excitatory neurons may be protective [3]. However, recent evidences draw a more complex scenario, in which alteration in the function of excitatory neurons and compensatory effects play important roles [14–17]. Elucidating the impact of *SCN1A* mutations in different cell populations and the consequences on brain function is relevant for the design of advanced therapies against DS. Recent efforts are focused on the increase of Na_v_1.1 production following different approaches [18]. Although a clear definition of the most suitable cell type for Na_v_1.1 restoration is still lacking, the general belief in the field is that preferential expression in inhibitory neurons could contribute to the safety and efficacy of the treatment.

Some therapeutic strategies leverage the existence of a healthy *SCN1A* allele as a source of functional Na_v_1.1. Antisense oligonucleotides (ASO) targeting a non-productive splicing event in the *SCN1A* gene result in an elevation of functional mRNA in animal models, with amelioration of the epileptic phenotype [19]. In principle, ASOs do not discriminate between different cell populations, but they act on the endogenous pre-mRNA, which is subjected to physiological transcriptional control. This approach (STK-001) is currently being evaluated in clinical trials sponsored by stoke therapeutics (MONARCH NCT04442295) and ADMIRAL ISRCTN99651026). Alternatively, artificial transcription factors (TF) specifically designed for the *SCN1A* locus can increase the Na_v_1.1 content in cells [20]. ETX101, developed by encoded therapeutics, is an adeno-associated vector (AAV) carrying a transcriptional activator for *SCN1A* under the control of a GABAergic neuron-specific promoter [21]. Initiation of the clinical trial is expected in 2023. If successful, this treatment offers the possibility of permanent improvement of the patient’s condition after a single vector administration.

On the other hand, the use of high-capacity adenoviral vectors (HC-AdV) allows gene supplementation approaches based on the delivery of the full *SCN1A* coding sequence (6 Kb), without the need of expressing artificial gene products [22]. In the original version, *SCN1A* expression was controlled by the potent, ubiquitous CAG promoter (early CMV enhancer, chicken β-actin promoter with first intron, and rabbit β-globin splice acceptor). Over-expression of *SCN1A* in a relatively low proportion of neurons and astroglia was well-tolerated in adolescent mice when the vector was injected in basal ganglia, pre-frontal cortex, and cerebellum. Increased survival and partial protection from hyperthermia-induced seizures was observed in a severe *Scn1a* A1783V *knock-in* mouse model [22]. However, the logical evolution of this approach after the initial proof of concept is the implementation of strategies to obtain a more physiological pattern of transgene expression.

In this work, we evaluate surface targeting and transcriptional control approaches to favour the expression of *SCN1A* in GABAergic neurons in the context of HC-AdV vectors. The adenoviral capsid was modified to incorporate ligands for proteins preferentially expressed in the target cells, such as ErbB4 [23] and Synaptotagmin II [24]. For the design of promoters, we evaluated regulatory sequences based on the complex and poorly defined *SCN1A* promoter [25–28], leveraging the extended cloning capacity of vectors. These regions were combined with other elements previously described to confer transcriptional specificity towards inhibitory neurons, such as the *VGAT* promoter [29] or the distal-less homolog (*Dlx*) enhancer [30].

## Materials and methods

### Plasmid construction

Firefly luciferase reporter plasmids were obtained by subcloning the different regulatory sequences into the pGL3-Basic backbone (Promega). Promoters from rat neuronal–specific enolase gene (*NSE*) [31], human PV, synapsin-2 (*Syn2*) [32], and vesicular GABA transporter (*VGAT*), as well as the human *Dlx* enhancer, were synthetized by GenScript. Fragments from the human *SCN1A* 5′ UTR were amplified by PCR from genomic DNA (Table 1), except for a 14-Kb fragment (F8) obtained from the BAC clone RP11-807A15 (Thermo Fisher Scientific). An intervening sequence (IVS) from the pIRES plasmid (Clontech) was placed between the *SCN1A*-based promoters and the firefly luciferase coding sequence in order to increase mRNA stability. Assembly of different combinations of sequences was performed using standard molecular biology techniques using restriction endonucleases from New England Biolabs. Ligase was from Promega, and oligonucleotides were purchased from Sigma-Aldrich. For the construction of the pAd-DP3V-GL plasmid, a cassette containing the DP3V promoter and the GFP-Luciferase fusion gene was inserted into the pSAdBst plasmid [22]. Genetic modifications in the fiber included a Y477A mutation in the knob to avoid CAR interaction [33] and the insertion of different targeting sequences in the HI loop (synthetized by GenScript). These sequences encoded the receptor binding domain from Clostridium Botulinum neurotoxin (residues 1113 to 1277) [34] or the EGF-like domain from neuregulin 1 alpha [35]. For the construction of the HC-AdV vectors, the corresponding expression cassettes (DP3V-Luc and DP3V-SCN1A) were introduced into the AscI sites of pD23-E4 [36] and pD20-E4 [22], respectively. pC-ErbB4-Neo is a bicistronic plasmid expressing mouse ErbB4 and the Neo^R^ genes under the control of the CMV promoter by virtue of an IRES upstream Neo^R^.

**Table 1:** Regulatory sequences used in this study. Indication of the chromosome (Chr), strand (Str), length (span), and location in the human genome (GRCh38/hg38 assembly), except for the rat *NSE* promoter (mRatBN7.2/m7 assembly)

**Name**	**Genomic coordinates**				
	**Chr.**	**Str.**	**Start**	**End**	**Span (bp)**
*SCN1A* F1	2	-	166.077.540	166.078.871	1332
*SCN1A* F2	2	-	166.073.622	166.074.911	1290
*SCN1A* F3	2	-	166.127.982	166.129.181	1200
*SCN1A* F4	2	-	166.149.124	166.151.673	2550
*SCN1A* F6	2	-	166.127.311	166.128.128	818
*SCN1A* F8	2	-	166.134.130	166.148.386	14257
*SCN1A* E2 enhancer	2	-	166.084.252	166.085.281	1030
*Dlx* enhancer	7	-	97.011.964	97.012.504	541
*VGAT* promoter	20	+	38.722.859	38.724.723	1865
*PV* promoter	22	-	36.822.572	36.824.108	1537
*NSE* promoter (rat)	4	-	157.580.887	15.758.2047	1161
*Syn2* promoter	3	+	12.003.540	12.004.551	1012

The *SCN1A* E2 refers to the human homolog of the mouse sequence previously described [48]

*Dlx* distal-less homolog, *NSE* neuronal-specific enolase, *PV* parvalbumin, *Syn2* synapsin-2, *VGAT* vesicular GABA transporter (*VGAT*)

### Vector production

The E1/E3-deleted Ad-CAG-GL vector (also known as Ad-CAG-GFPLuc) has been previously described [22]. The Ad-DP3V-GL vector was obtained using the same method, by transfection of the pAd-DP3V-GFPLuc plasmid in HEK-293 cells. The culture was maintained until cytopathic effect (CPE) was apparent. The virus was cloned by end-limiting dilution and amplified in HEK-293 cells. Purification was performed by double CsCl density gradient ultracentrifugation and desalting by buffer exchange using Amicon Ultra Centrifugal Filters‐Ultracel 100 K (Millipore). Final buffer formulation was Tris 100 mM with 10% glycerol. Production of HC-AdVs was performed as previously described [37]. Quantification in viral genomes (vg) was performed by qPCR of genomes isolated from purified virus [38] using the primers 5′ AGCATCCGTTACTCTGAGTTGG 3′ (forward); GCATGTTGGTATGCAGGATGG (reverse) for E1/E3-deleted vectors, and 5′ TAGTGTGGCGGAAGTGTGATGTTG 3′ (forward); 5′ ACGCCACTTTGACCCGGAACG 3′ (reverse) for HC-AdVs.

### Cell culture

The cell lines HEK-293 (American Type Culture Collection, ATCC CRL-1573), Neuro2A (ATCC CCL-131), and U87 (ATCC HTB-14) were maintained in DMEM. Hygromycin (0.1 mg/ml, Life Technologies) was added to 293Cre244 cells. The 293-ErbB4 cell clone was established by transfection with the pC-ErbB4-Neo plasmid and selection with geneticin (G418), 0.4 mg/ml (Sigma). SH-SY5Y (ATCC CRL-2266) was maintained in a 1:1 mixture of Eagle’s Minimum Essential Medium and F12 Medium supplemented with 1% non-essential amino acids. THP-1 cells (ATCC TIB-202) were maintained in RPMI-1640 Medium supplemented with 0.05 mM 2-mercaptoethanol. Differentiation to macrophages was carried out by treatment with 0.5 M phorbol myristate acetate (PMA) for 48 h (Sigma) [39]. All culture media were supplemented with 10% foetal bovine serum (FBS), 100 U/ml penicillin, 100 μg/ml streptomycin, and 2 mM L-glutamine. Reagents were obtained from Gibco. All cells were maintained at 37 °C with 5% CO_2_ in a humidified incubator. Cells were routinely tested for mycoplasma contamination.

The basic protocol for GABAergic neuron differentiation was described by Franchi et al. [40]. The medial ganglionic eminence (MGE) was dissected from C57BL6/J embryos (E14.5) and incubated in Hank’s balanced salt solution (HBSS, Life Technologies) supplemented with 10 mM HEPES (Gibco). Cell dissociation was performed by adding 30 µL of DNase I at 10 mg/ml (Roche) and 60 µL of 2.5% trypsin solution (Gibco), followed by a 15-min incubation at 37 °C. Enzymatic digestion was stopped by adding 5 ml of plating medium (neurobasal medium (Gibco) supplemented with 10% FBS (Sigma), 2% B-27 (Thermo), 1% GlutaMAX (Gibco), 100 U/ml penicillin, and 100 μg/ml streptomycin (Gibco)). Then, the mixture was centrifuged at room temperature (RT) for 5 min at 90 g, the supernatant was removed, and 2 ml of maintenance medium (MM: neurobasal medium (Gibco) supplemented with 2% B-27 (Thermo), 1% GlutaMAX (Gibco), 100 U/ml penicillin, and 100 μg/ml streptomycin (Gibco)) was added to carry out the mechanical dissociation. Cells were seeded at a density of 2–3 × 10^5^ cells per well on 24-well plates (Eppendorf). Plates were coated with 100 ug/ml poly-L-lysine (Sigma) diluted in sterilized double distilled water and 2.5 mg/ml Matrigel (Corning, 374,244) diluted in Leibovitz’s L-15 Medium (Gibco). Neurons were maintained in 800 µL of MM, and BDNF (50 ng/ml, PeproTech) was added 12 h later.

Astrocytes and microglia primary cultures were obtained from newborn mice. T-75 culture flasks were coated with 0.5 ml of 200 µg/ml poly-D-lysine (Sigma) for 1 h at 37 °C. Brains were placed into a Petri dish containing 5 ml of (DMEM)-high glucose GlutaMAX (Gibco). Meninges, blood vessel and cerebellum were removed under a dissection microscope. The remaining tissue was minced into small pieces and transferred into a 50-ml tube containing 2.5 ml of an enzymatic solution (116 mM NaCl, 5.4 mM KCl, 26 mM NaHCO_3_, 1 mM NaH_2_PO_4_, 1.5 mM CaCl_2_, 1 mM MgSO_4_, 0.5 mM EDTA, 6.25 μL L-Cysteine, 11.25 mg glucose, 24 μL papain and 0.325 μL DNase I) and incubated at 37 °C for 10 min. Next, the content was filtered through a 70-µm cell filter, homogenized in a 15-ml tube with 5 ml of 20% heat-inactivated FBS in HBSS and centrifuged at 1.500 rpm for 5 min. After resuspending and homogenizing the pellet in 9 ml of DMEM supplemented with 10% FBS, 100 U/ml penicillin, 100 µg/ml streptomycin and 5 ng/ml of granulocyte-macrophage colony-stimulating factor (GM-CSF), the cell suspension was placed in the T-75 poly-D-lysine-coated flask. The next day, cell debris was removed by washing with PBS, and media was replaced. The process was repeated every 3–4 days. In approximately 7 days, astrocytes form a confluent cell layer at the bottom of the flask. Microglia grows on top of the astrocyte layer, and it takes 7 additional days to reach optimal confluency to be isolated. The flask was shaken at 150 rpm for 3 h, and the supernatant, containing the microglia, was collected and centrifuged at 1.500 rpm for 5 min. After adding 10 ml of DMEM plus antibiotics to the flask, it was shaken again at 150 rpm to remove residual microglia. Next day, the media was removed, cells were washed twice with PBS, and then, they were trypsinized (Gibco). Finally, astrocytes were seeded at a density of 1.5 × 10^5^ cells/cm^2^ into poly-D-lysine-coated 24-well plates (Eppendorf).

### Transfections, infections, and luciferase assay

Cells lines were seeded in 24-well plates at 80–90% confluency. Transfection was performed 24 h later by Lipofectamine 2000 (Invitrogen) using 0.5 μg of each Firefly luciferase reporter plasmid. The transfection mixture was removed 5 h later and replaced by fresh culture medium. Primary cells were transfected at the end of the differentiation process using the same method. Cells were lysed 48 h later, and 10 μL of each sample were analysed for luciferase activity using the Luciferase Reporter Assay System (Promega) on a Lumat LB 9507 Luminometer (Berthold Technologies). Results were normalized by microgram of protein loaded, determined by Bradford assay (BioRad). Infection of cell lines and primary cells was carried out in 24-well plates seeded with 2 × 10^5^ cells. Viral load (multiplicity of infection (MOI) was defined as vg/cell. Cell lysates were obtained 48 h after infection for quantification of luciferase activity or GFP fluorescence (flow cytometry). In the case of GABAergic neurons, identification of cells showing GFP fluorescence was carried out by microscopy because they do not withstand the processing for flow cytometry.

### Flow cytometry analysis

The percentage of transduced cells was determined by analysis of GFP expression. To this end, cells were trypsinized from the 24-well plate, collected, and centrifuged at 2.500 rpm for 3 min at 4 °C. Cell pellets were resuspended in 200 μL of paraformaldehyde 2% (Electron microscopy sciences) for 5 min at 4 °C and centrifuged at 2.000 rpm for 3 min. Supernatants were discarded, and pellets were resuspended in 200 μL buffer autoMACS (Sigma) supplemented with 1% FBS, 100 U/ml penicillin, 100 μg/ml streptomycin, and 2.5 mM EDTA (Sigma). Finally, cells expressing GFP were quantified in a FACS CANTO cytometer (10.000 events per sample).

### Quantitative PCR

Cell pellets were processed with the Maxwell^®^ 16 LEV simplyRNA Cells/Tissue Kit (Promega) for total RNA isolation following manufacture’s indications. Two micrograms of RNA were then treated with DNase I and retro-transcribed into cDNA using M-MLV retro-transcriptase enzyme (Invitrogen) and random primers (Life Technologies). These procedures were performed in a GeneAmp^®^ PCR System 2400 (Applied Biosystems). Quantitative analysis was performed by real-time PCR using a iQTM SYBR^®^ Green Supermix reagent (Bio-Rad) in CFX96 Touch^™^ Real-Time PCR Detection System (Bio-Rad). Primers were purchased from Sigma (mouse *Gad65*: FP 5′ CTCTGCTCTCCTGGTTAGA 3′ and RP 5′ AACTATGGCTGATGTGGAG 3′; human *GAD65* FP 5′ CTCTGCTCTCCTGGTTAGA 3′ and RP 5′ CTCCACATCAGCCATAGTT 3′; mouse and human *VGLUT*: FP 5′ GCTGTGTCATCTTCGTGAGG 3′ and RP 5′ CAGGCGACTCCGTTCTAAGG 3′; mouse and human *VGAT*: FP 5′ CATCCAGGGCATGTTCGTG 3′ and RP 5′ AGGCACGCGATGAGGATC 3′; mouse *Scn1a*: FP 5′ CATGTATGCTGCAGTTGATTCCA 3′ and RP 5′ AACAGGTTCAGGGTAAAGAAGG 3′; human *SCN1A* FP 5′ TCAACATGTACATTGCCGTC 3′ and RP 5′ ATCAGCTGCAGTTTGTTGG 3′. The relative quantification was carried out using the 2^−∆Ct^ method [41] using mouse/human 36b4 as housekeeping gene (FP 5′-AACAATCTCCCCCTTCTCCTT-3′ and RP 5′-GAAGGCCTTGACCTTTTCAG-3′).

### Analysis of transduction in vivo

#### Stereotactic injection

Five-week-old C57BL/6 J mice (Envigo) were anesthetized with ketamine/xylazine (80:10 mg/kg, i.p.) and placed in a stereotactic frame. After shaving and disinfecting the scalp, a longitudinal incision was made along the midline to expose the skull, which was cleaned with iodine and hydrogen peroxide to remove the periosteum and prevent infection. Next, bilateral burr holes were drilled at HC coordinates (anteroposterior −1.94, mediolateral ± 1.20, and dorsoventral −2.00 mm) determined according to the Paxinos and Watson mouse brain atlas (1998). A 10-µl Hamilton syringe (Hamilton Co) was used for vector delivery. Viral suspensions (1 × 10^8^ vg in 1.0–1.5 µl/injection) were administered following a 0.4-µl/min infusion rate. The incision was stitched, and the animals were observed until full recovery from anaesthesia. Injection in the thalamus is described in the section of therapeutic evaluation below.

#### Bioluminescence imaging (BLI)

The substrate D-luciferin (REGIS Technologies) was administered intraperitoneally (200 µl of a 33.3 µg/µl solution in PBS) to anesthetized mice. Light emission was detected 5, 10, 15, and 30 min later using a PhotonImager^™^ Optima apparatus (BioSpace) for identification of the peak values. Data were analysed using the M3Vision software (BioSpace), representing the maximal value obtained for each animal.

#### Immunofluorescence (IF) procedures

Animals were perfused transcardially with 0.9% saline under an overdose of ketamine/xylazine anaesthesia (240:30 mg/kg body weight). Brains were removed, fixed in 4% paraformaldehyde (PanReac) for 24 h at RT, and then cryopreserved in 30% sucrose solution in PBS at 4 °C until they sank. Microtome sections (thickness: 30 μm) were cut with a freezing microtome and stored in cryopreserving solution (30% ethylene glycol (Sigma), 30% glycerol (Sigma) in PBS) at −20 °C until processed. IF was performed in 2–4 free-floating tissue sections per animal. Brain sections were washed 3 times with PBS at RT, and then, a blocking step was performed (2% donkey or goat normal serum (Jackson Immuno Research), 0.5% Triton X-100 (Sigma), and 1% BSA (Sigma) in PBS), followed by overnight incubation at 4 °C with the primary antibodies (rabbit anti-GFP, Abcam, Cat# MAB377, 1:5.000; chicken anti-GFP, Abcam; Cat# ab13970, 1:5.000; mouse anti-GFAP (GA5), Cell Signaling Technology, Cat# 3670, 1:500; mouse anti-VGlut1, 1:250, Sigma, Cat# AMAb91041; rabbit anti-GAD65/67, 1:200, Abcam, Cat# EPR19366; and rabbit anti-Calretinin, 1:1000, Chemicon, Cat# AB5054) diluted in REAL antibody diluent (Agilent). After washing 3 times with PBS, slices were incubated with the secondary antibodies (donkey anti-Rabbit IgG (H + L) Alexa Fluor 488 Cat# A-21206, 1:400; goat anti-mouse IgG (H + L) Alexa Fluor 568 Cat# A-11031, 1:400); and goat anti-chicken IgY (H + L) Alexa Fluor 488 Cat# A-150173, 1:400) for 90 min at RT. To enable the visualization of nuclei, sections were incubated for 5 min with the DNA marker 4′,6-diamidino-2-phenylindole (DAPI, Thermo Fisher Scientific Cat# D1306, 300 nM) and washed twice with PBS. Finally, slices were mounted on super frost plus slides (Thermo Fisher Scientific), air-dried for 24 h, and rinsed in toluene (5 min). Coverslip (Thermo) were placed with Immu-Mount^®^ mounting medium (Thermo Fisher Scientific). Fluorescence signals were acquired with the Axio Imager.M1 microscope (Zeiss) using the EC Plan Neofluor 20x/0.50 M27 objective. Images were acquired with the AxioCam MR R3 camera and the program ZEN 2 blue edition (Zeiss). Acquired fluorescence images were adjusted in parallel for brightness and contrast in ZEN 2 blue edition (Zeiss). Quantifications were performed by two independent blinded investigators using the *ImageJ* software.

### Evaluation of therapeutic effect in the DS model

*Scn1a*-A1783V mice (B6(Cg)-*Scn1a*tm1.1Dsf/J, The Jackson Laboratory, stock no. 026133) were bred to mice expressing Cre recombinase under the control of the CMV promoter (B6.C-Tg(CMV-Cre)1Cgn/J, The Jackson Laboratory, stock no. 006054 [42]), as previously described [12, 43, 44]. This conditional knock-in mouse model is referred hereinafter as *Scn1a*^WT/A1783V^ or DS mice. Vectors were injected at the age of P21-24 in mice anesthetized by ketamine/xylazine (191/4.25 mg/kg). Carprofen (5 mg/kg) was used for analgesia. After placing mice in a stereotaxic device (Ultra Precise Stereotaxic Instruments, Stoelting), a midline incision was made above the skull, and holes were made using a 25-G needle. The vectors were delivered to the thalamus and hippocampus. To that end, the needle was first lowered to AP −1.8 mm; ML ± 1.8 mm; DV −3.5 mm, and a volume of 0.5 $\mu$l (0.5 × 10^8^ vg) was injected at a rate of 100 nl/min (Quintessential Stereotaxic Injector, Stoelting, Wood Dale, IL, USA). The needle was kept in place for ~5 min; then, the injection needle was raised to DV 3 mm; and another volume of 0.5 $\mu$l was injected. Sensitivity to hyperthermia-induced seizures was analysed using a chamber equipped with a heat lamp (TCAT-2DF, Physitemp Instruments). Mice were placed in the chamber 10 min before heating, and the baseline body temperature was measured using a rectal probe. Temperature was increased 0.5 °C every 2 min until seizure started or body temperature reached 40.5 °C.

### Statistical analysis

The *GraphPad* Prism software was used for analysis. Data sets following normal distribution were compared using one-way ANOVA with Dunnett’s multiple comparisons tests (more than two groups), or *T*-test (two groups). Otherwise, groups were compared using Kruskal-Wallis with Dunn’s multiple comparison test (more than two groups) or Mann-Whitney test (two groups). Correlations were analysed by linear regression. Survival and percentage of seizure-free mice were analysed by Log-Rank test.

## Results

### A hybrid regulatory sequence containing elements from the *Dlx*, *SCN1A*, and *VGAT* genes (DP3V) promotes preferential transgene expression in GABAergic neurons

**Fig. 1:**
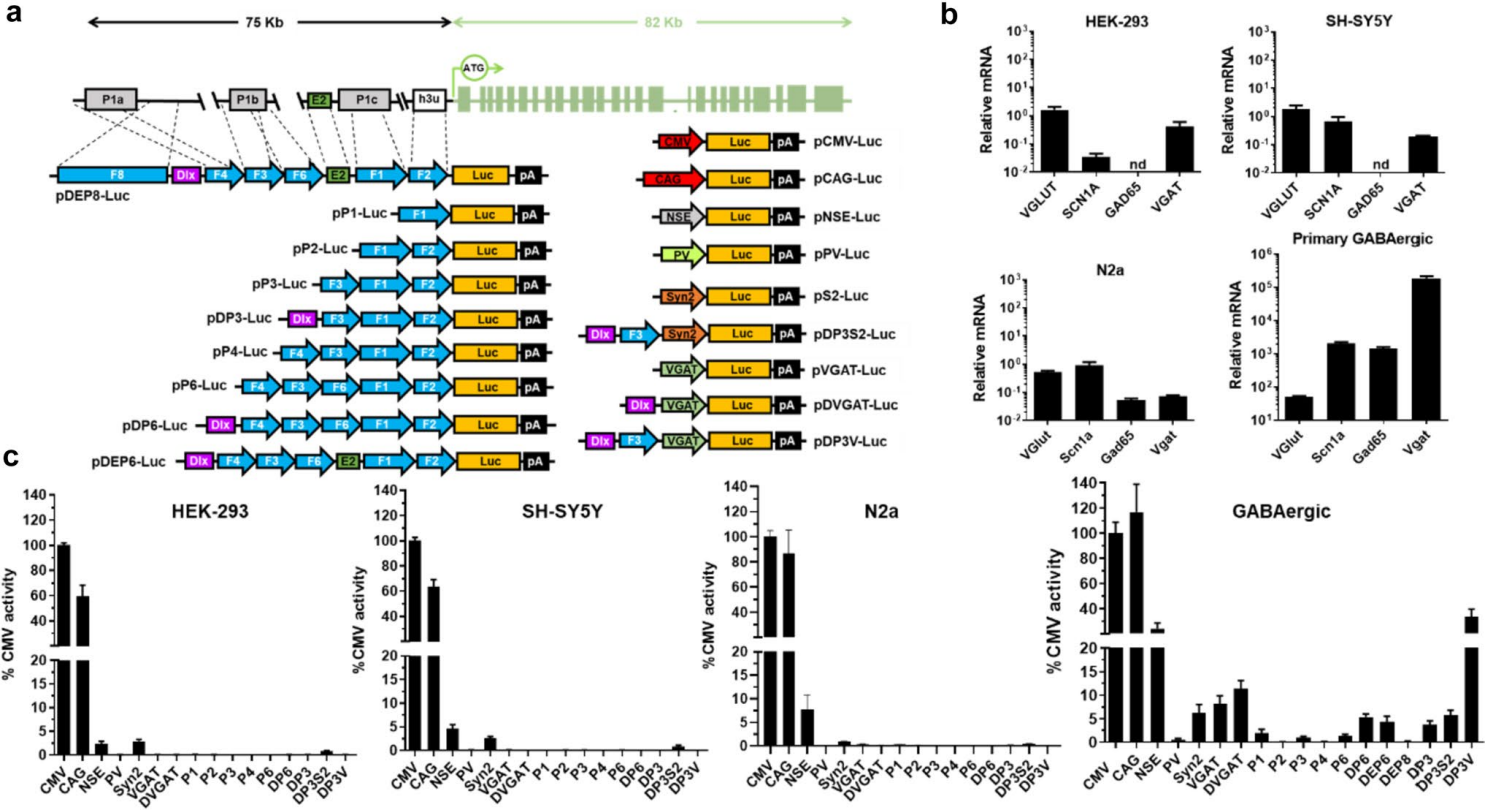
Identification of regulatory sequences with preferential activity in GABAergic neurons. **a** Schematic representation of luciferase reporter plasmids (not drawn to scale). Approximate correspondence of sequences based on the human *SCN1A* 5′UTR (named as F1–F8) with putative regulatory regions (P1a–c promoters, gray boxes [25–27, 45–47]), E2 enhancer (green box) [48] and h3u untranslated exon (white box) [46]. The coding exons downstream the translational start site (ATG) are depicted as green boxes. **b** Expression of the indicated endogenous genes in different cell lines, analysed by qRT-PCR. The graph shows the relative mRNA content, using the 36b4 gene as a reference (2^ΔCt^). nd, not detected. Values are multiplied by 1.000 for easier visualization. **c** Luciferase activity in cells transfected with the indicated reporter plasmids, expressed as percentage relative to pCMV-Luc

The sequences controlling the expression of *SCN1A* are complex and only partially defined [25–28]. The 5′ region presents different transcriptional start sites (TS) in a sequence extending more than 75 Kb from the initiation of translation. A recent bioinformatic study contributed to define the boundaries of the regulatory regions and proposed 3 promoters (P1a-c) [45]. In addition, seven enhancers were predicted around 250 Kb from the initiation of translation, and two additional ones in an extended 700 Kb region. Of note, human and mouse sequences present a high degree of synteny and 70% homology. Based on previous reports and in silico predictions, we selected different regions from the human *SCN1A* and cloned them upstream of the luciferase reporter gene (Fig. 1a; Table 1). The panel of regulatory sequences included promoters of genes highly expressed GABAergic neurons such as PV, synapsin-2 (*Syn2*), and vesicular GABA transporter (*VGAT*) [29, 32], as well as the pan-neuronal promoter derived from the rat neuronal-specific enolase gene (*NSE*) [31]. Finally, the CAG and the human cytomegalovirus immediate-early promoter/enhancer (CMV) are representative ubiquitous promoters.

For the evaluation of these sequences, the luciferase reporter plasmids were transfected in the widely used cell line HEK-293 (derived from an immature neuronal precursor in the human embryonic kidney [49]), SH-SY5Y (human neuroblastoma), and Neuro-2a (N2a, mouse neuroblastoma). This group is representative of immature, undifferentiated cells with neuronal origin. We were unsuccessful in obtaining reliable differentiation of cell lines to GABAergic neurons following different protocols. In contrast, we managed to obtain this phenotype from primary cultures obtained from the MGE of mouse embryos [40]. Expression of GABAergic markers was verified by qRT-PCR, in contrast with the immature cells (Fig. 1b). As expected, the promoter activity of CMV and CAG was high in all cells (Fig. 1c). CMV was used as a reference for comparison of promoter strength across different cells. The *NSE* promoter had relatively low but measurable activity in immature neurons (2–7% on average compared with CMV), and close to 25% in GABAergic neurons. The *Syn2* promoter followed a similar pattern, with lower intensity (2% in immature cells and 6% in GABAergic neurons). Surprisingly, the *PV*-derived sequence and all regions from the *SCN1A* 5′UTR showed negligible activity in all cells, including GABAergic neurons. In contrast, the *VGAT* promoter demonstrated the expected specificity in the target cells, with 8% activity compared with CMV. Considering these results, we designed hybrid promoters based on *VGAT*. Addition of the *Dlx* enhancer to the *VGAT* promoter (DVGAT) caused no significant variation in its activity on GABAergic cells. Interestingly, when the P3 region of the *SCN1A* gene was inserted between *Dlx* and *VGAT* (DP3V promoter), the activity reached 30% in the target cells, without significant increase in immature neurons. Combination of the *Dlx* enhancer with the P3 or P6 regions showed a moderate elevation of luciferase activity in GABAergic cells. Finally, we assembled a sequence containing the *Dlx* enhancer and a combination of *SCN1A* regions: the P6 region, an additional 14-Kb-long region from the gene (F8), and the human homolog of a recently described enhancer (E2) preferentially active in PV-expressing interneurons (see Table 1 for details) [48]. However, this large construct (DEP8) showed low activity in GABAergic cells.

### Targeting of adenoviral vectors to the ErbB4 receptor does not confer preferential transduction of GABAergic neurons

**Fig. 2:**
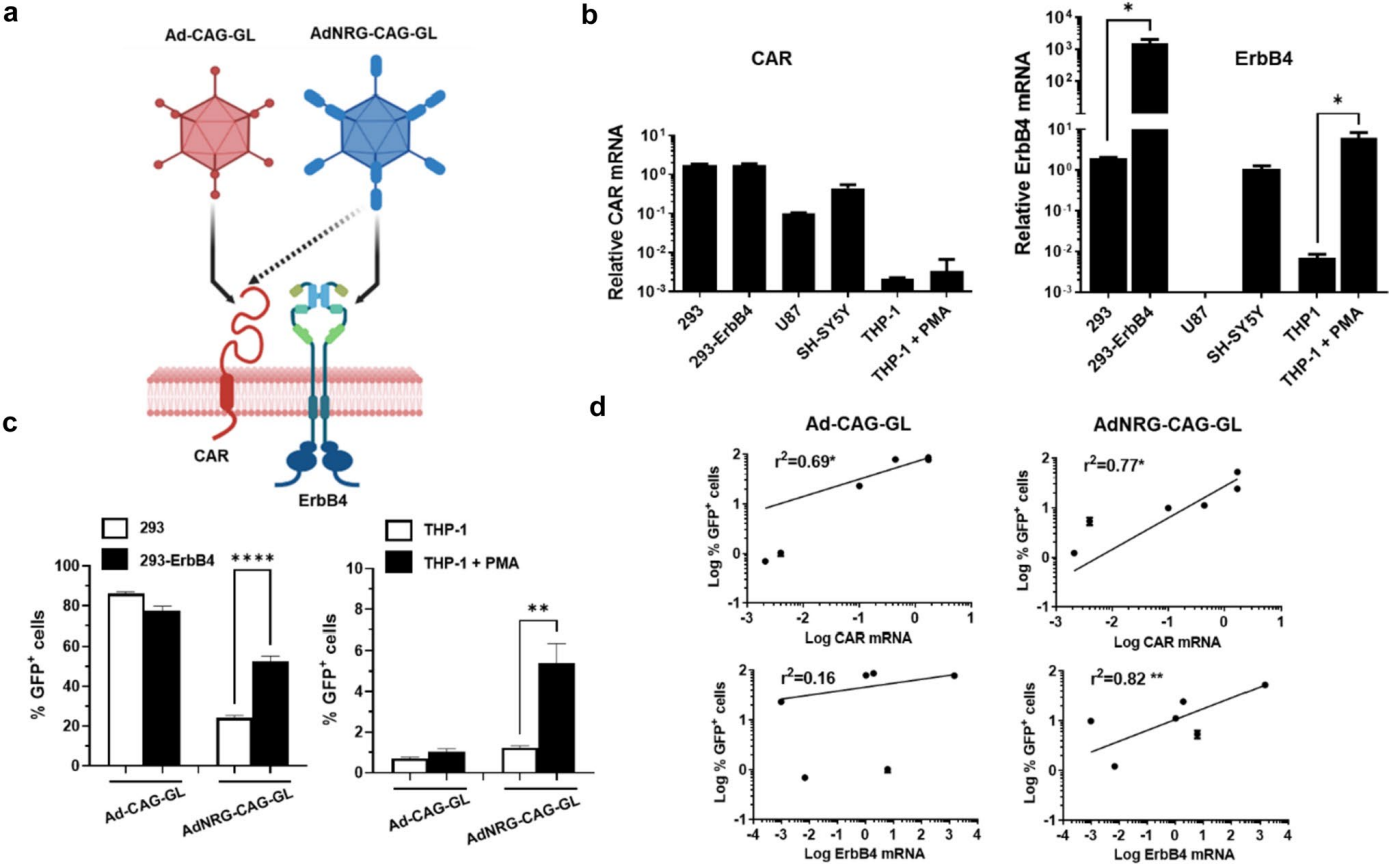
Incorporation of the EGF-like domain from NRG1α into the HI loop of adenovirus fiber favours transduction of cells expressing ErbB4. **a** Schematic representation of capsid modifications introduced in the AdNRG-CAG-GL vector and the expected impact on infectivity. Created with BioRender.com. **b** Expression of the receptors CAR and ErbB4 in different cell lines, analysed by qRT-PCR. A subset of THP-1 cells were treated with 0.5 mM PMA. The graph shows the relative mRNA content, using the 36b4 gene as a reference (2^ΔCt^). Values are multiplied by 1.000 for easier visualization. **c** The indicated cells were infected with Ad-CAG-GL or AdNRG-CAG-GL at MOI 10 (vg/cell), and 48 h later the percentage of cells showing GFP fluorescence was quantified by flow cytometry. *p < 0.05; **p < 0.01; ****p < 0.0001. **d** Correlation of infectivity (% of GFP^+^ cells) and CAG or ErbB4 expression in a panel of cell lines. *p < 0.05; **p < 0.01, linear regression

We selected ErbB4 and synaptotagmin-2 (*Syt2*) as potential receptors for targeting adenoviral vectors to PV-positive GABAergic interneurons, based on the location of these proteins in the surface of the cells and the availability of well-defined ligands (Fig. 2a). On one side, Syt2 is a synaptic vesicle membrane protein preferentially expressed by inhibitory neurons in the brain, with good correlation with PV expression [24]. In contrast with other members of the Syt family, Syt2 is transiently exposed to the extracellular space. Clostridium Botulinum neurotoxin from serotype B (BoNT/B) binds to Syt2 through its receptor binding domain (RBD) located in the C-terminal portion of its heavy chain (Hcc) [34]. This interaction triggers the internalization of the toxin to the endosomal membranes and then to the cytosol of neurons. Therefore, we hypothesize that attachment of the RBD to the HI loop of the AdV fiber could result in preferential infection of Syt2-expressing cells in the brain. However, this capsid modification (involving incorporation of a 165 aa-long peptide) was not compatible with viral production, despite the use of specific packaging cells over-expressing Syt2 (data not shown).

On the other side, NRG1 belongs to a family of neurotrophic factors that bind ErbB4 through their EGF-like domains [23]. It has been previously described that incorporation of the domain from NRG1α (52 aa) into the HI loop of AdV fiber allowed preferential transduction of ErbB4-expressing cells in vitro [50], confirming that this modification is compatible with the correct assembly of capsids. In order to reduce the natural binding of adenovirus to the coxackie and adenovirus receptor (CAR, which is widely expressed in neurons), we introduced the Y477A mutation in the fiber knob [33]. We produced an E1/E3-deleted adenoviral vector harbouring these capsid modifications and expressing the GFPLuc reporter gene under the control of the CAG promoter (AdNRG-CAG-GL). The viral yield was low compared with an equivalent vector with wild type HAdVC5 capsid, even in packaging cells over-expressing ErbB4 (6 × 10^10^ and 2.8 × 10^11^ vg/ml for AdNRG-CAG-GL and Ad-CAG-GL, respectively). For initial in vitro characterization, we used a panel of cell lines with different levels of ErbB4 and CAR expression (Fig. 2b). Since the infectivity of adenoviral vectors depends on the pattern of expression of different primary and secondary receptors, the most straightforward comparison is between pairs of cells which differ mainly in the expression of ErbB4. By flow cytometry analysis of GFP expression, we observed that the capsid-modified vector AdNRG-CAG-GL transduces more efficiently HEK-293 over-expressing ErbB4 than the parental cells (Fig. 2c). In contrast, Ad-CAG-GL shows no preference. The same behaviour was reproduced when we compared the human monocytic cell line THP-1 in basal conditions and after differentiation to macrophages by PMA treatment (Fig. 2c), which increases ErbB4 expression (Fig. 2b). Next, we analysed the correlation of infectivity (percentage of GFP^+^ cells) and the expression of either CAR or ErbB4 in a larger panel of cell lines (Fig. 2d). As expected, Ad-CAG-GL showed positive correlation only with CAR expression. In contrast, the infectivity of AdNRG-CAG-GL was associated with the expression of both receptors. These results indicate that the display of the EGF-like domain in the capsid of Adenovirus favours transduction of cells expressing high levels of ErbB4.

**Fig. 3:**
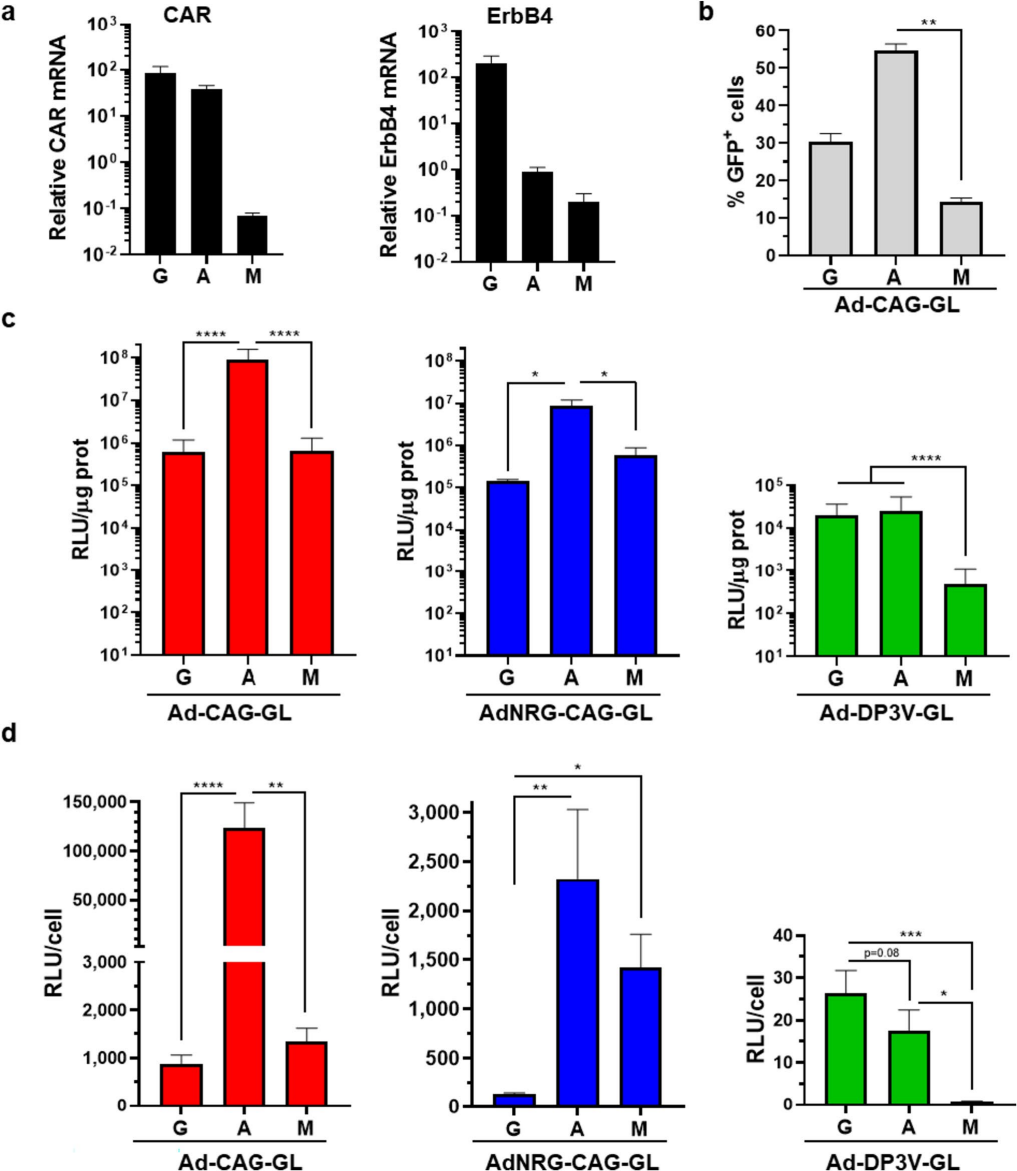
The Ad-DP3V-GL vector, but not AdNRG-CAG-GL, facilitates transgene expression in primary GABAergic neurons. Primary cultures of GABAergic neurons (G), astrocytes (A), or microglia (M) were obtained from C57BL/6 J mice. **a** Expression of the receptors CAR and ErbB4 in differentiated cells, analysed by qRT-PCR. The graph shows the relative mRNA content, using the 36b4 gene as a reference (2^−ΔCt^). Values are multiplied by 1.000 for easier visualization. Cells were infected with vectors at MOI 100 and were analysed 48 h later. **b** Cells infected with Ad-CAG-GL were processed for flow cytometry (astrocytes and microglia) or examined under a fluorescence microscope (GABAergic neurons) to determine the percentage of cells showing GFP fluorescence. Cells infected with the indicated vectors were lysed to measure luciferase activity in cell extracts relative to protein content (**c**), and normalized by transduction efficacy (**d**). *p < 0.05; **p < 0.01; ***p < 0.001; ****p < 0.0001 Kruskal-Wallis with Dunn’s post-test

However, the mutation of the CAR-binding domain in the fiber knob (Y477A) does not completely abrogate binding and infection through this receptor. This circumstance, together with the existence of other co-receptors such as integrins, heparan sulphate proteoglycans, and scavenger receptors, prevents a reliable prediction of vector infectivity in different cell populations. Therefore, we analysed this parameter in mouse GABAergic neurons, astrocytes and microglia primary cultures. Despite consistent expression of CAR and ErbB4 in GABAergic neurons (Fig. 3a), the parental vector Ad-CAG-GL achieved higher percentage of GFP^+^ cells in astrocytes, whereas transduction of microglia was very low (Fig. 3b).

**Fig. 4:**
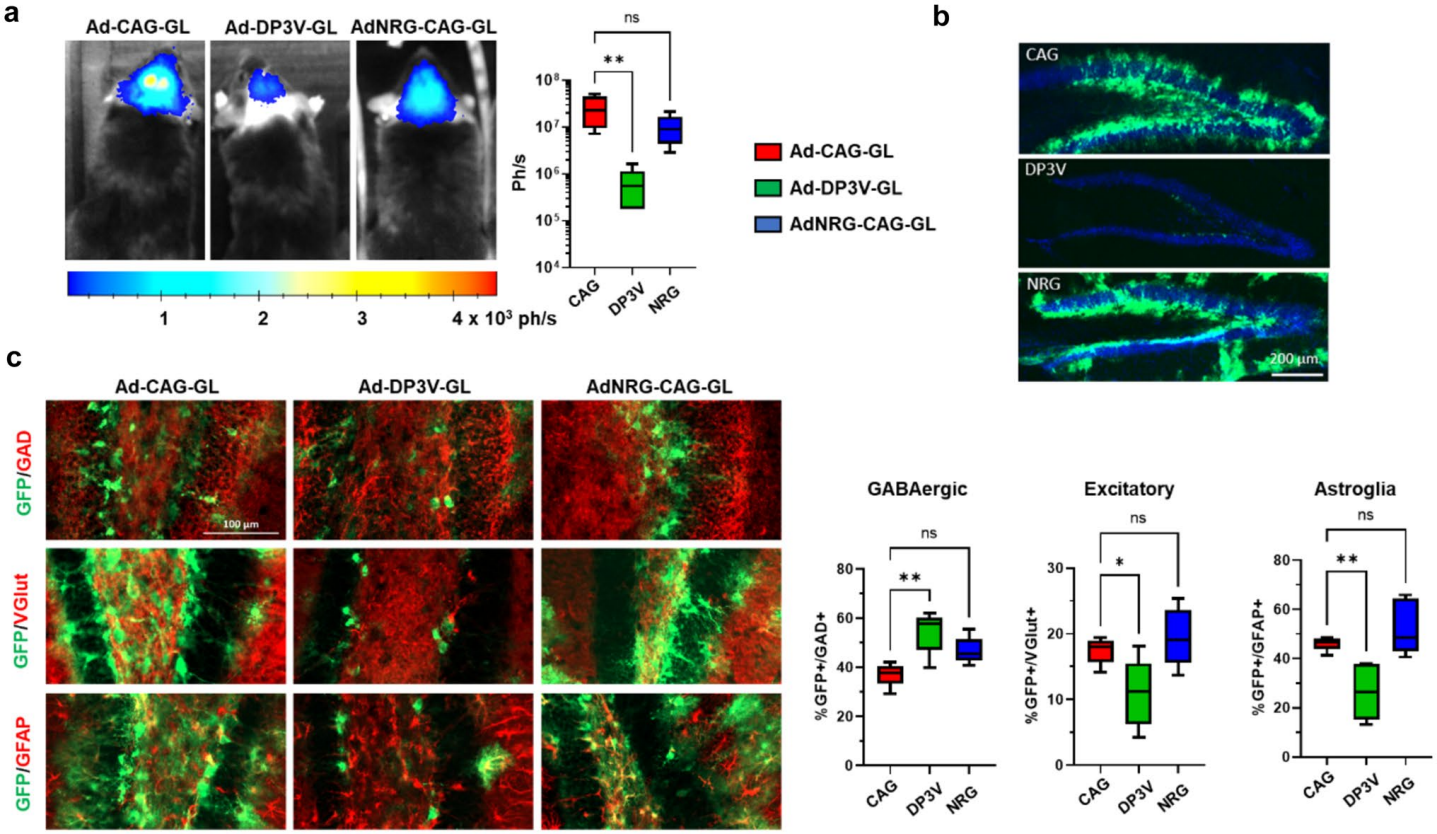
The Ad-DP3V-GL vector, but not AdNRG-CAG-GL, shows preferential transgene expression in GABAergic neurons in vivo. The indicated vectors were injected in the HC of C57BL/6 J mice (1 × 10^8^ vg/injection, bilaterally), *n* = 5. Forty-eight h later, light emission from the head was quantified by BLI, and then, brains were processed for IF. **a** BLI images from representative mice (left) and light emission quantification (in photons/second). **b** Low magnification images of the dentate gyrus from representative mice injected with the indicated vectors. GFP expression is marked in green, and nuclei are stained with DAPI (blue). **c** Representative images of brain slices co-stained for GFP (green) and the indicated markers in red (GAD65/67 for GABAergic neurons, VGlut1 for excitatory neurons, and GFAP for astroglia). The percentage of GFP^+^ cells expressing each marker is shown in the graphs on the left. *p < 0.05; **p < 0.01, ANOVA with Dunnett’s post-test

The analysis of luciferase activity in cell extracts confirmed that Ad-CAG-GL induces higher transgene expression in astrocytes compared with GABAergic neurons and microglia (Fig. 3c). Surprisingly, AdNRG-CAG-GL showed the same pattern. This result indicates that promotion of ErbB4 binding though capsid modification does not achieve preferential transduction of primary GABAergic neurons. To study if the vectors showed the same behaviour in vivo, they were administered by stereotactic injection in the hippocampus (HC) of C57BL/6 mice (1 × 10^8^ vg/injection, bilateral). Forty-eight hours later, mice were sacrificed and brains were processed for IF analysis. We determined the percentage of transduced (GFP^+^) cells displaying markers of GABAergic neurons (GAD65/67), excitatory neurons (VGlut), or astroglia (GFAP). As shown in Fig. 4, no significant differences were detected when the patterns of transduction of AdNRG-CAG-GL and Ad-CAG-GL were compared.

### Transcriptional targeting using the hybrid DP3V promoter achieves preferential transgene expression in GABAergic neurons

After the failure of surface targeting, we focused on the transcriptional control of gene expression. The Ad-DP3V-GL vector incorporates the GFP-Luc reporter gene under the control of the DP3V promoter, in the context of a wild-type HAdVC5 capsid. As expected from the information generated with reporter plasmids (Fig. 1c), primary cultures infected with Ad-DP3V-GL showed low luciferase activity in general (Fig. 3c), but the pattern was different from Ad-CAG-GL. Despite the high infectivity of adenovirus in astrocytes, transgene expression upon infection with Ad-DP3V-GL was equivalent to GABAergic cells. When light emission was corrected by infectivity (RLU/GFP^+^ cell), the preferential activity of the DP3V promoter in GABAergic neurons became evident, especially compared with microglial cells (Fig. 3d). The difference with excitatory neurons or astroglia was confirmed in the in vivo experiment (Fig. 4). When the Ad-DP3V-GL vector was injected in the HC of mice, light emission was lower than in the case of Ad-CAG-GL or AdNRG-CAG-GL (Fig. 4a), and the number of cells expressing GFP was also restricted (Fig. 4b). Interestingly, cells expressing GFP upon infection with the Ad-DP3V-GL vector were preferentially GABAergic (GAD65/67^+^), whereas the percentage of excitatory neurons or astroglia was reduced in comparison with Ad-CAG-GL (Fig. 4c). Preferential expression in GAD65/67^+^ cells was also observed when Ad-DP3V-GL was administered in the thalamus of mice (Supplementary Fig. 1). In contrast, we found that the transduction of Calretinin (CR)^+^ cells showed no significant difference between Ad-CAG-GL and Ad-DP3V-GL (Supplementary Fig. 2). Interestingly, the expression of *SCN1A* in this sub-population of GABAergic cells is very low or absent [3].

### A HC-AdV vector expressing *SCN1A* under the control of the DP3V promoter shows therapeutic effect in a mouse DS model

**Fig. 5:**
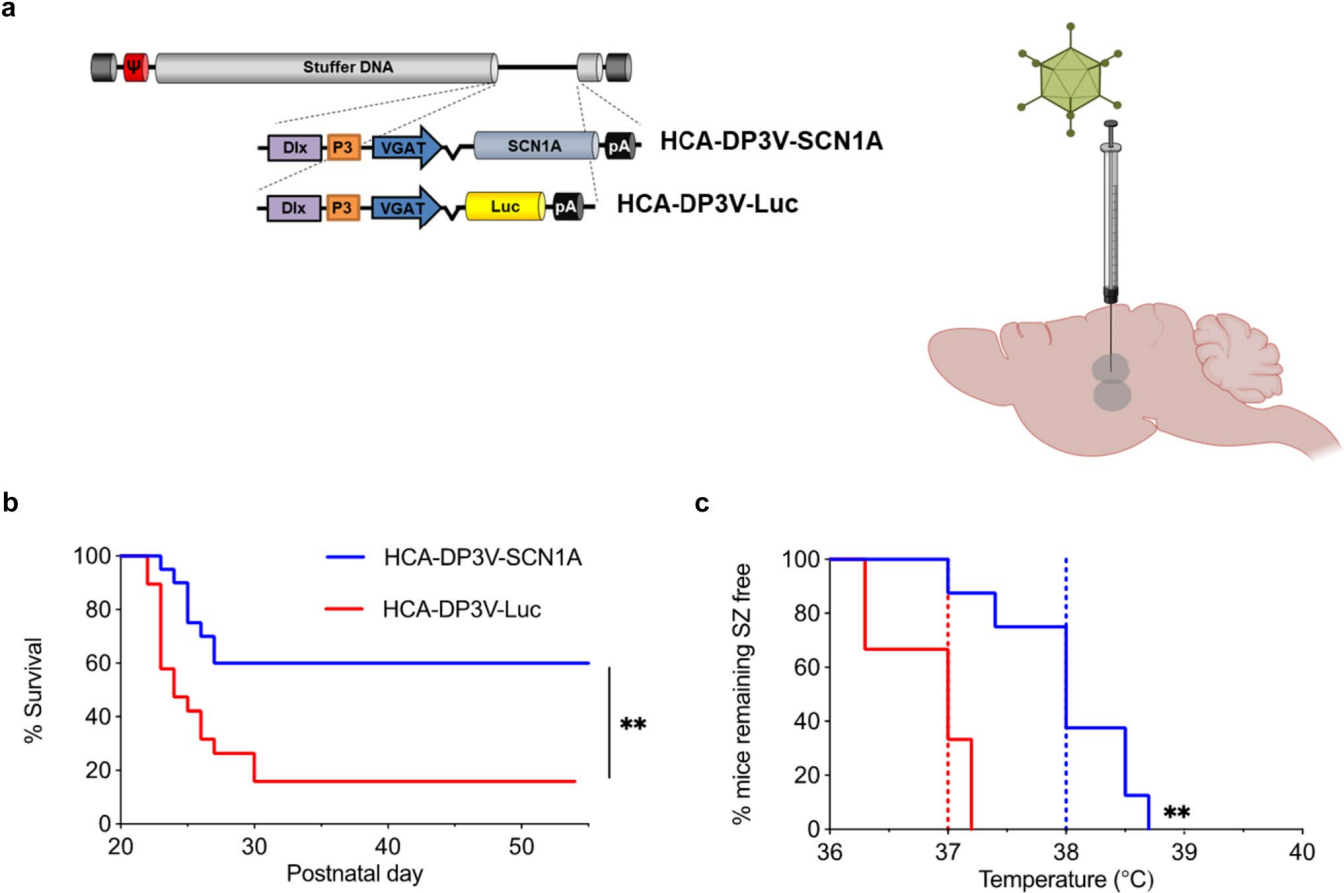
Preferential expression of *SCN1A* in GABAergic cells improves survival and epileptic phenotype in a DS mouse model. **a** Schematic representation of HC-AdVs used in the study and their administration route. Created in part with BioRender.com. **b** Survival curve of DS mice injected with either HCV-DP3V-Luc (*n* = 19) or HCA-DP3V-SCN1A (*n* = 20) at P21-P24. **c** Four weeks after treatment, surviving mice were subjected to hyperthermia. The graph shows the percentage of mice remaining free of thermally-induced seizures (sz). HCA-DP3V-Luc (*n* = 3); HCA-DP3V-SCN1A (*n* = 8). The dotted lines represent the median seizure temperature. DS: **p < 0.01 log-rank test

The DP3V promoter was used to control the expression of a codon-optimized version of the *SCN1A* coding sequence, and the expression cassette was inserted in a HC-AdV vector (HCA-DP3V-SCN1A). The vector was administered bilaterally in the HC and thalamus of 3 weeks-old *Scn1a*^WT/A1783V^ mice by stereotactic injection (1 × 10^8^ vg/injection). An equivalent vector expressing luciferase instead of *SCN1A* (HCA-DP3V-Luc) was used as a control (Fig. 5a). The treatment was well tolerated, and we observed a significant increase of survival in the group of mice treated with the therapeutic vector (Fig. 5b). To evaluate the epileptic phenotype, mice were subjected to controlled hyperthermia 4 weeks after treatment. A significant increase in the seizure threshold temperature was detected in mice treated with HCA-DP3V-SCN1A (Fig. 5c). These results demonstrate that preferential expression of *SCN1A* in inhibitory neurons at moderate levels has therapeutic effect in a DS model.

## Discussion

*SCN1A* is among the genes most frequently associated with epilepsy and genetic encephalopathies, and it might contribute to age-related cognitive decline [5]. Therefore, the interest in methods to enhance *SCN1A* expression is growing in the neurosciences field [18–22]. Each disease may require specific adaptations to restore the altered Na_v_1.1 function. In the case of loss of function (LOF) mutations causing DS, the most accepted hypothesis is that *SCN1A* upregulation should take place preferentially in inhibitory neurons [3]. In gene supplementation approaches using adenoviruses, redirecting vector tropism can restrict transgene expression to specific cells, but this approach is challenging because the capsid is a complex structure. On one side, not all modifications are compatible with viable virions, as we have experienced with the addition of the BoNT/B RBD into the HI loop of fiber. On the other hand, infection depends on a combination of primary and accessory receptors, which explains the wide tropism of adenoviruses. Although addition of the EGF-like domain from NRG1α favoured infection of cells expressing high levels of ErbB4, this was not sufficient to obtain specific infection of primary GABAergic neurons in cell culture or in vivo. Probably, the affinity of the modified fiber knob for the canonical CAR is still higher than the added NRG1α domain, as described in previous attempts to target cancer cells [50]. A powerful alternative to rational design of capsid modifications is the in vivo screening of vector libraries. This approach is being successfully employed to change the tropism of AAV vectors [51], but it can be adapted to more complex particles such as adenoviruses.

Regarding transcriptional targeting, the best option would be to obtain physiological control of transgene expression using the endogenous promoter, which would obviate the need for previous definition of target cells. However, all regulatory sequences based entirely on the *SCN1A* 5′ UTR showed very low transcriptional activity in our experiments. We opted for a hybrid sequence in which fragments from *VGAT*, *SCN1A*, and *Dlx* promoters cooperated to achieve specific activity in GABAergic cells. The Dlx5/6 and *VGAT* promoters are active in all GABAergic cells, according the studies performed in knock-in mice [52, 53]. In contrast, *SCN1A* expression is low or absent in some subpopulations such as CR^+^ cells [3]. The cooperative function of the three elements may be needed for the optimal activity of the DP3V promoter. This could explain why the Ad-DP3V-GL vector showed no preferential transgene expression in CR^+^ cells. During the preparation of this manuscript, a bioinformatic study proposed a more precise location of functionally relevant regulatory regions in the *SCN1A* gene [45]. Three alternative promoters were defined: P1a (GRCh38.p13: 166.148.180–166.151.550), P1b (166.127.360–166.129.030), and P1c (166.077.140–166.079.490). Interestingly, the most active transcriptional start site (TSS) in the brain is located in P1b (166.128.014), which is included in the F3 fragment of DP3V. This 3.6-Kb-long hybrid promoter added to the *SCN1A* cDNA requires vectors with high cloning capacity (at least 9.6 Kb). Our results demonstrate the suitability of HC-AdVs, but other options such as lentiviruses, herpes viruses, or non-viral vectors could be considered.

The *SCN1A* mutation in our DS model and most DS patients affects all cells, and the physiological expression of this gene is not exclusive to GABAergic neurons [1]. However, our results indicate that preferential expression of transgenic *SCN1A* in this cell population shows therapeutic effect, despite the relatively low potency of the promoter. Comparison between the efficacy of ubiquitous versus GABAergic neuron-specific expression of the transgene is beyond the scope of this work. On the one hand, this objective would require a promoter with stronger specificity than DP3V. On the other hand, extensive dose-response studies should be performed in the DS model. Moderation in the amount of transgenic Na_v_1.1 produced in transduced cells may contribute to increase the safety of the therapy. Whereas it is not clear that over-expression of wild type *SCN1A* is functionally equivalent to gain of function (GoF) mutations, high transgene expression could alter protein homeostasis in cells, with potential neuronal damage in the long-term. Other approaches recently developed for Na_v_1.1 augmentation cause discrete elevations in expression and are designed to be active in cells already producing the *SCN1A* mRNA (STK-001), or in GABAergic neurons (ETX101). Here, we describe the first vector for long-term expression of transgenic *SCN1A* preferentially in GABAergic neurons, without the need for concomitant expression of non-human proteins. In summary, we have increased the repertoire of gene therapy vectors designed against DS and describe a strategy that may lead to further refinements in gene supplementation approaches for this disease.

### Supplementary Information

Below is the link to the electronic supplementary material.

Supplementary file1 (DOCX 1140 KB)

## Data Availability

All data generated during this study are included in this published article.
