# Supplementary figures and images for: Preferential expression of SCN1A in GABAergic neurons improves survival and epileptic phenotype in a mouse model of Dravet syndrome
Source: J Mol Med (Berl). 2023 Oct 11;101(12):1587–601. doi: 10.1007/s00109-023-02383-8 (PMC10697872; doi:10.1007/s00109-023-02383-8)

**
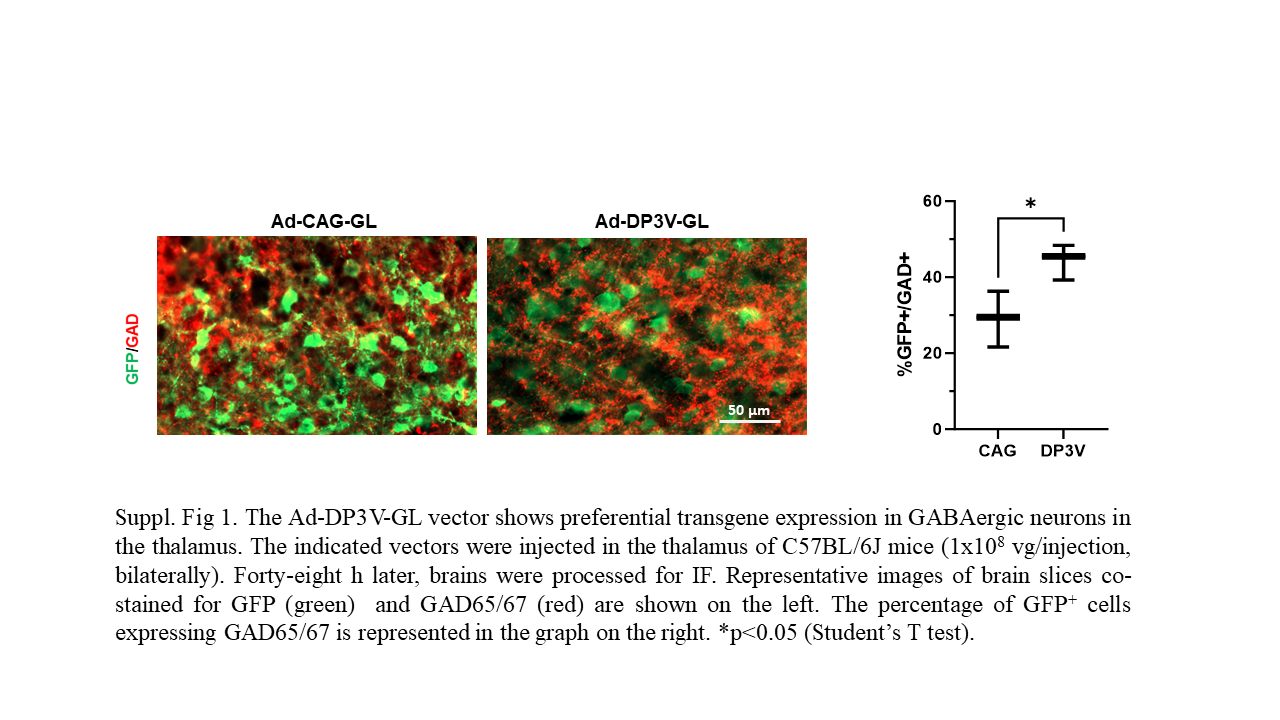

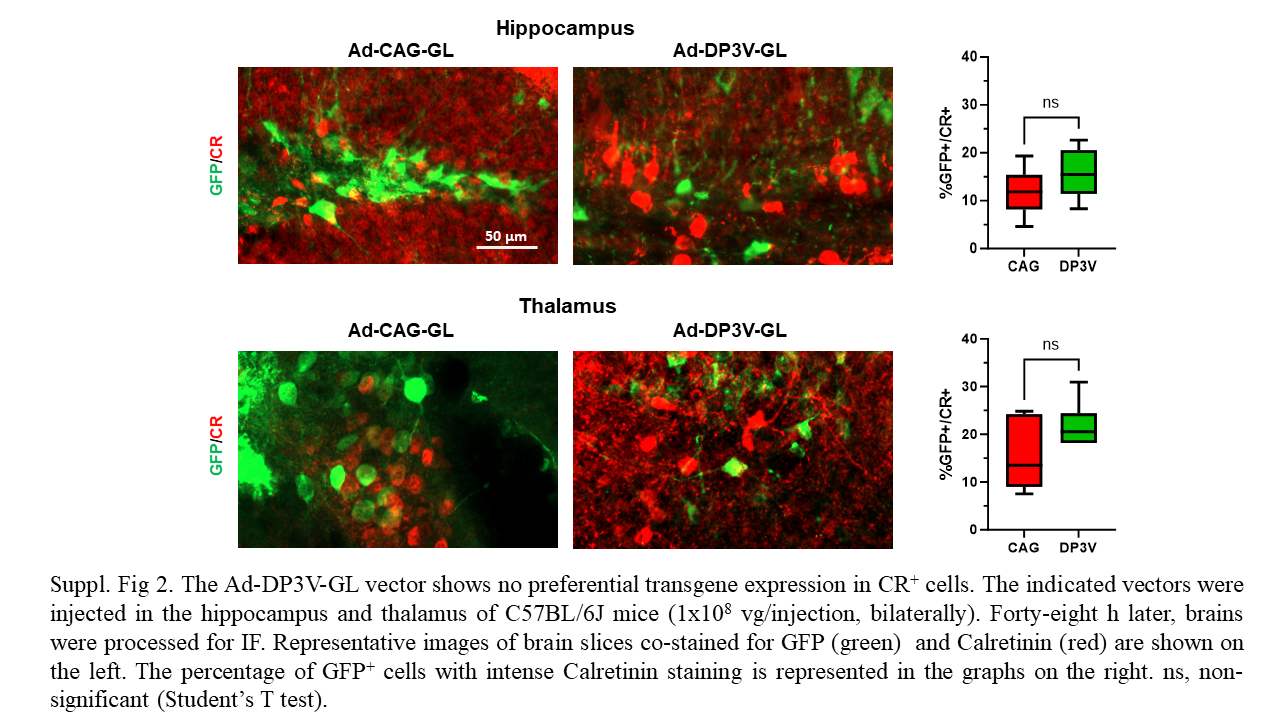
**

Supplement: Supplementary file 1 — Supplementary file1 (DOCX 1140 KB) [file 109_2023_2383_MOESM1_ESM.docx]
